# Lake water volume fluctuations in response to climate change in Xinjiang, China from 2002 to 2018

**DOI:** 10.7717/peerj.9683

**Published:** 2020-08-20

**Authors:** Adilai Wufu, Hongwei Wang, Yun Chen, Yusufujiang Rusuli, Ligang Ma, Shengtian Yang, Fei Zhang, Dan Wang, Qian Li, Yinbo Li

**Affiliations:** 1 College of Resource and Environmental Science, Xinjiang University, Urumqi, Xinjiang Uygur Autonomous Region, PR China; 2 CSIRO Land and Water, Canberra, Australia; 3 Institute of Geographical Science and Tourism, Xinjiang Normal University, Urumqi, Xinjiang Uygur Autonomous Region, PR China; 4 College of Water Sciences, Beijing Normal University, Beijing, PR China

**Keywords:** Lake volume change, Arid region, Large scale, Altitude, Altimetry data

## Abstract

Climate change has a global impact on the water cycle and its spatial patterns, and these impacts are more pronounced in eco-fragile regions. Arid regions are significantly affected by human activities like farming, and climate change, which influences lake water volumes, especially in different latitudes. This study integrates radar altimetry data from 2002 to 2018 with optical remote sensing images to analyze changes in the lake areas, levels, and volumes at different altitudes in Xinjiang, China. We analyzed changes in lake volumes in March, June, and October and studied their causes. The results showed large changes in the surface areas, levels, and volumes of lakes at different altitudes. During 2002–2010, the lakes in low- and medium-altitude areas were shrinking but lakes in high altitude areas were expanding. Monthly analysis revealed more diversified results: the lake water levels and volumes tended to decrease in March (−0.10 m/year, 37.55×10^8^ m^3^) and increase in June (0.03 m/year, 3.48×10^8^ m^3^) and October (0.04 m/year, 26.90×10^8^ m^3^). The time series lake water volume data was reconstructed for 2011 to 2018 based on the empirical model and the total lake water volume showed a slightly increasing trend during this period (71.35×10^8^ m^3^). We hypothesized that changes in lake water at high altitudes were influenced by temperature-induced glacial snow melt and lake water in low- to medium-altitude areas was most influenced by human activities like agricultural irrigation practices.

## Introduction

Inland lakes are important water resources in arid and semi-arid areas (Schindler, 2009; Williamson, Saros & Schindler, 2009; Zhao et al., 2017), contributing to the sustainable development of the social economy and the protection of the local ecology. Climate change greatly impacts water resources in arid and semi-arid areas like Xinjiang, China (Dilinuer & Aikebaer, 2010; Zhang et al., 2019b; Zhang et al., 2015b). Water resources are key to the development of social and economic activities and watershed construction in Xinjiang, due to its fragile ecosystem. Xinjiang’s water resources are becoming increasingly scarce due to global climate change and excessive human economic activities (Zhang et al., 2013) leading to rapid shrinkage (Bian et al., 2006; Zhang et al., 2015a), salinization (Zuo et al., 2006) and even the complete drying up of lakes (Sarp & Ozcelik, 2016; Wang, Gong & Shao, 2014), seriously endangering the ecological environment and their adjacent areas. Studies of the change in water resources in Xinjiang are limited, especially in a time series aspect and a large spatial scale.

Xinjiang is an extremely arid region of China in the center of the Eurasian continent, far from the sea (Li et al., 2011). Inland lakes are important water resources in Xinjiang and directly affect and influence the ecological environment of this vast territory of complex geological structures and large topographic variations. Many different types of lakes exist in this region due to its varied geomorphology and climate but these lakes have undergone changes since the mid-20th century, which is concerning to many experts and scholars. Remote sensing water indexes have been used frequently to assess the varying water levels (Zhang et al., 2015a; Ziyinali, 2018). However, previous studies (Dai et al., 2020; Wang et al., 2019; Yang et al., 2019; Zhang et al., 2015a) primarily focused on the changes in the surface areas and water levels of one or more lakes. Research on water volume estimation and its variations has been neglected in arid and multi-geomorphological regions and in-depth analysis of time series and characteristic monthly aspects has not been conducted.

Water volume is a key indicator of the water budget and cannot be calculated directly (Crétaux et al., 2017). Traditional water volume estimation methods are based on the data of shore topography maps and water level or the inversion model of lake depth data (Medina et al., 2010; Peng, Guo & Liu, 2006). However, these approaches are inefficient, and time- and energy-consuming, especially in places like Xinjiang where climate conditions are harsh and conventional hydrological observations are inadequate. Previous studies have estimated water volume changes based on satellite altimetry and optical image data (Baup, Frappart & Maubant, 2014; Busker et al., 2019; Chipman, 2019; Zhang, Chen & Xie, 2019a; Zhang et al., 2013). Recent studies have focused on lake surface areas (Ji et al., 2015; Khandelwal et al., 2017) and levels (Ricko et al., 2012; Song et al., 2015; Wang et al., 2011), but few have focused on volume (Medina et al., 2010; Qiao, Zhu & Yang, 2019). Few studies have been conducted measuring long-term water volume changes, especially in different latitudes and large-scale arid areas that take into account climate change and human activities (Frappart et al., 2018; Khaki et al., 2018). Additional studies are needed to determine the significant factors affecting changes in lake water volume with respect to different latitudes.

We sought to combine altimetry data with remotely-sensed images to analyze the water volume changes and the climate response of different lakes at various altitudes in Xinjiang from 2002 to 2018. Three important issues were investigated: (1) estimation of the lake surface areas, levels, and volumes based on altimetry data and optical remote sensing image; (2) exploration of the spatial and temporal pattern of lake surface areas, levels, and volumes at different altitudes; and (3) the examination of the controlling factors of water changes at high altitudes.

### Study area

**Figure 1:**
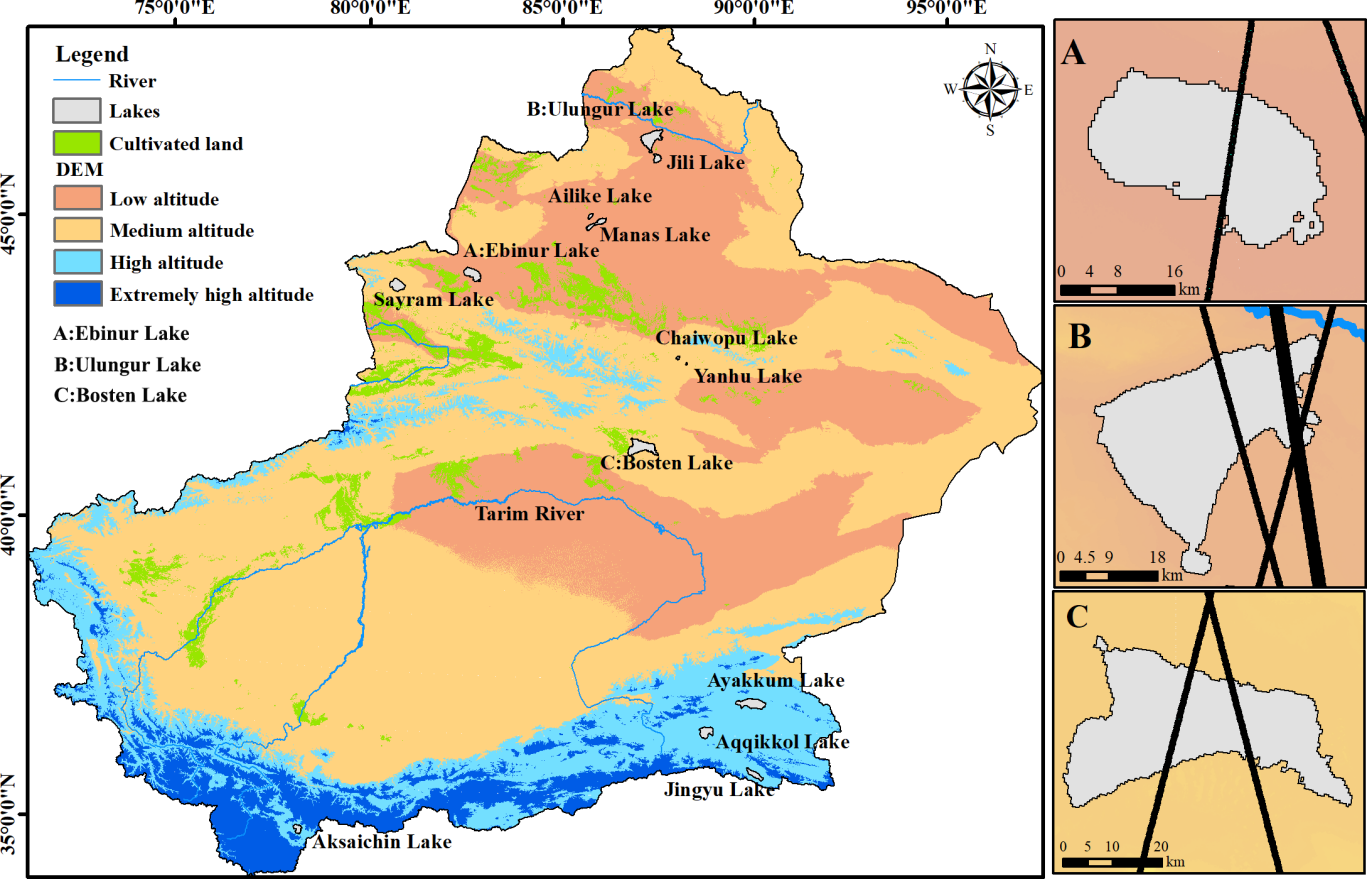
Lakes distribution and altimetry tracks. (A) Ebinur Lake and Envisat tracks; (B) Ulungur Lake and ICEsat tracks; (C) Bosten Lake and Envisat tracks.

Xinjiang is an arid and semi-arid region located between 34°15′−49°10′N and 73°20′−92°25′E in north-western China with an area of 1.67 ×10^6^ km^2^(Fig. 1). High-elevation mountains, including the Altai Mountains in the north of Xinjiang, the Kunlun Mountains in the south, and the Tianshan Mountains in the middle of Xinjiang, divide Xinjiang into Northern and Southern Xinjiang. The mean annual precipitation of the area ranges between 100 and 200 mm (Wang et al., 2012). Xinjiang is far from the ocean and has a continental arid and semi-arid climate with an annual average temperature varying from 4 to 8 °C in Northern Xinjiang and from 10 to 13 °C in Southern Xinjiang. Vegetation is sparse in the plains and the altitudinal belts of the mountains are obvious. Xinjiang is characterized by desert and bare lands due to a lack of water. The ecological environment is highly vulnerable. Our study area can be categorized into four levels according to the altitude classification standard of low-altitude (<1000 m), medium-altitude (1000–3500 m), high-altitude (3500–5000 m), (Zhou et al., 2010) and extremely-high-altitude (>5000 m).

### Data

We used the MOD09A1 8-day composite and 500-m resolution land surface reflectance product (Vermote, 2015) to estimate lake surface areas (https://ladsweb.nascom.nasa.gov/search/). Some bodies of water were frozen from November to February due to a lack of snow melt, which may cause inaccuracies in the water area extraction. Therefore the lakes were analyzed annually in March, June, and October to identify the seasonal patterns of the lakes. We used the MOD09A1 data for the 89th, 153rd, and 273th days of 2002–2010 over Xinjiang to assist in determining seasonal patterns, which represented March 29th, June 3rd, and October 3rd, respectively.

Altimeter datasets from Envisat/RA-2 and ICESat/GLAS were used to obtain water levels. Envisat/RA-2 data (Gardini, Graf & Astronautica, 1995) has 35-day temporal resolution guarantees, providing observations of the Earth’s surface from 82.4° (lat, N) to 82.4° (lat, S). Envisat Level 2 provides four algorithms designed specifically to extract geophysical measurements over different types of surfaces: Ocean re-tracker, Ice-1 re-tracker (Wingham, Rapley & Griffiths, 1986), Ice-2 re-tracker (Legrésy & Remy, 1997) and Sea-Ice re-tracker (Laxon, 1994). Ice-1 was used in this study for its proven accuracy for land hydrology purposes (Frappart et al., 2018; Medina et al., 2010) (https://earth.esa.int/web/guest/missions/esa-operational-eo-missions/envisat). ICESat/GLAS data (Schutz et al., 2005; Zwally et al., 2002) were taken two or three times a year from 2003 to 2009 and 19 instances of campaign data were obtained (Wang et al., 2011). 15 types of GLAS data were provided for scientific uses (Zwally et al., 2002) and GLA14 was used to determine the water levels of lakes from 2003–2009 (https://nsidc.org/data/icesat).

Landsat-7 ETM+ data was selected from the summer of 2010 as reference data to validate the accuracy of the water extracted from MODIS data. The 13 lakes were covered by 10 Landsat images taken close to the date of MODIS and all of the images show less than 5% cloud cover (http://www.gscloud.cn/). We obtained the monthly in-situ water level data of Bosten Lake from the Bazhou Hydrological and Water Resource Bureau to validate the water level data from satellite altimetry and to conduct linear regression analysis. We referred to the spatial interpolation dataset of annual average temperature and annual precipitation provided by Chinese Academy of Sciences Resource and Environment data cloud platform to obtain the time series temperature and precipitation information (http://www.resdc.cn/lds.aspx). We analyzed data from the years 2002 to 2015 in section: “The increase of water volume in high altitude area”. The data was not updated after 2015.

### Method

#### Surface area change

Researchers have proposed a variety of methods for water extraction using remote sensing techniques (Davranche, Lefebvre & Poulin, 2010; Frappart et al., 2006; Hanqiu, 2007; Lei, Li & Bruce, 2009; Ouma & Tateishi, 2007) and SVM was frequently well documented (Sarp & Ozcelik, 2016; Tong et al., 2016). We used the support vector machine (SVM) for lake surface area extraction using MODIS data. The SVM algorithm is a supervised, non-parametric machine-learning technique based on structural risk minimization strategies that reduce misclassification errors for the training set (Pal & Foody, 2010). SVMs perform well with a limited number of training samples and are able to find complex land cover patterns in high-dimensional feature sets (Mountrakis, Im & O, 2011). SVM is a linear classifier in the parameter space and can be extended as a nonlinear classifier by the use of the kernel function. The accuracy of SVM classification largely depends on the determined kernel function. An SVM classifier includes four different types of kernels: linear, polynomial, radial basis function, and sigmoid. In this study, the radial basis function was selected as the kernel method for SVM classification. This function works well in most cases and can handle linearly non-separable problems.

MOD09A1 data has seven bands that are corrected for atmospheric and other conditions such as atmospheric scattering, ozone, aerosol, clouds, bi-directional reflection, and sun elevation. Previous studies determined that bands 2, 5, 6, and 7 were all well-suited for extracting water surface and band 2 was optimal for small water bodies (Wu & Zhang, 2005). All bands of MODIS were composited for the purpose of performing supervised classification in our study. The body of water was shown in black on the composited remote sensing images and other surface types were bright, giving a sharp contrast and playing an important role in the water body extraction.

**Figure 2:**
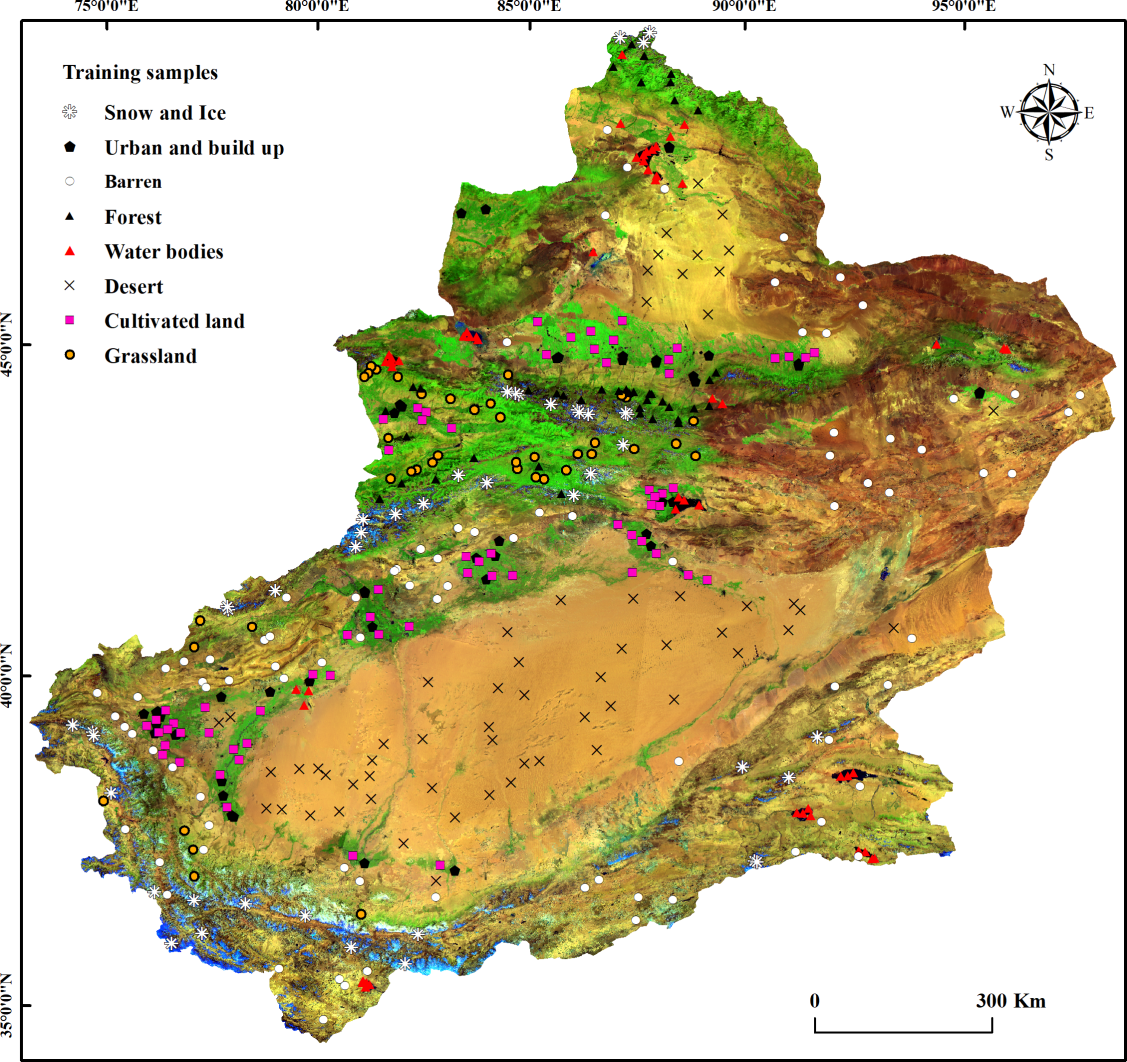
Locations of the training samples used for classification.

Training data are needed to execute SVM. 386 training samples were selected in the time-series MODIS images. The samples were classified into 8 categories including: water bodies, urban and build up, grassland, forest, cultivated land, desert, snow and ice and barren, and performed SVM classification. The classification training samples were collected at random from the representative homogeneous areas and the locations of the training samples are shown in Fig. 2. Then the classification results were merged into two categories: water and non-water, and manual editing and accuracy validation were performed with reference to Landsat and Google Earth images.

### Surface height calculation

The lake level corresponded to the distance between the lake surface and the geoid. The extraction method consisted of four steps: Step 1: For each satellite track that intersects with a lake, precisely select the footprints (elevation) within the lake using SVM classification results; Step 2: Extract the parameters of each footprint including time, altitude, range, a series of error corrections and geoid; Step 3: Refine the point selections by removing the outliers; Step 4: Cross-analyze Envisat and ICESat results and the constant offsets were calculated as the mean difference between the different altimeters. The Envisat data was used as reference and the system bias was subtracted to all the ICESat results to eliminate the constant offsets between Envisat and ICESat. The ICESat dataset was then projected to TOPEX/Poseidon ellipsoid and the Envisat data was projected to WGS84. We unified the data by converting the ICESat dataset to the WGS84 ellipsoid, (Bhang, Schwartz & Braun, 2007) using Eq. (1):

(1) \begin{eqnarray*}\mathrm{ICESat}\text{_}\mathrm{elevation}=\mathrm{ICESat}\text{_}\mathrm{elevation}\text{_}\mathrm{measured}-\mathrm{ICESat}\text{_}\mathrm{geoid}-0.7\end{eqnarray*}

Where the ICESat_elevation_measured and ICESat_ geoid was directly provided from the ICESat data and the 0.7 m is the offset from Topex ellipsoid to WGS84 ellipsoid.

### Accuracy assessment for water surface area and water level

The accuracy of the water surface area extracted from MODIS was validated by high-resolution Landsat images. Band 754 of Landsat ETM+ images were composited and supervised classification was performed using the SVM method. The classification results of Landsat and MODIS were compared from the perspectives of spatial distribution and the lake area. The in situ measured water level was used to validate the accuracy of water level testing obtained by altimetry data and was used for a linear regression analysis.

### Modeling changes in water volume

The water volume of lakes with an irregular surface area was calculated according to the volume of the round table. As the water level rises, the surface area expands and the water volume increases; as the water level drops, the surface area shrinks and the corresponding amount of water volume decreases. Therefore, the changes of water volume of lakes can be obtained according to changes in the water surface area and water level. The formula used is as follows (Taube, 2000) Eq. (2):

(2) \begin{eqnarray*}\Delta V= \frac{1}{3} \times \left( {S}_{1}+{S}_{2}+\sqrt{{S}_{1}\times {S}_{2}} \right) \times \Delta H\end{eqnarray*}

Where Δ*V* represents the water volume change during two periods, *S*_1_ and *S*_2_ represent the water surface area of two periods, and Δ*H* represents the change of water level during two periods.

### Extrapolation method

Although the accuracy of satellite altimetry is high, a serious problem in detecting water level change is its discontinuous nature and the short-time span of time-series elevation data, which lasted only from 2003 to 2010. Corresponding empirical models were developed based on the relationship between the surface area and water level of lakes to measure time-series elevation data for different lakes to gather long-term data of the water levels. The water volume changes of the lakes between 2011 and 2018 were estimated by combining the time series water surface area, the water level, and the aforementioned algorithm.

## Results and Analysis

### Accuracy assessment

#### Surface area

**Figure 3:**
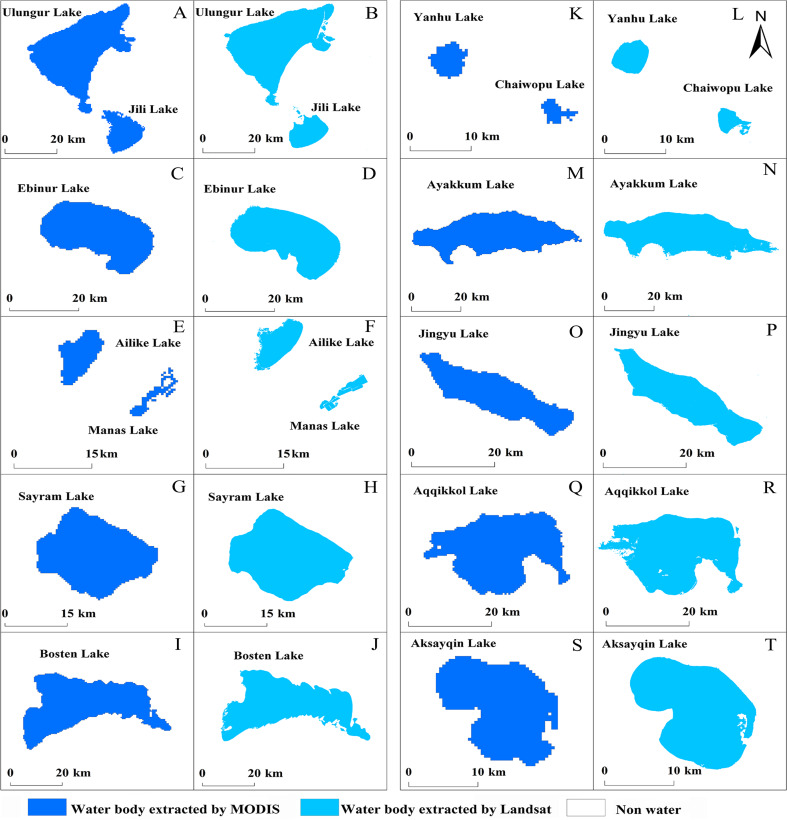
Comparison of lake surface area extracted from two different images. (A-F) Low-altitude lakes; (G-L) medium-altitude lakes; (G-L) high-altitude lakes.

**Figure 4:**
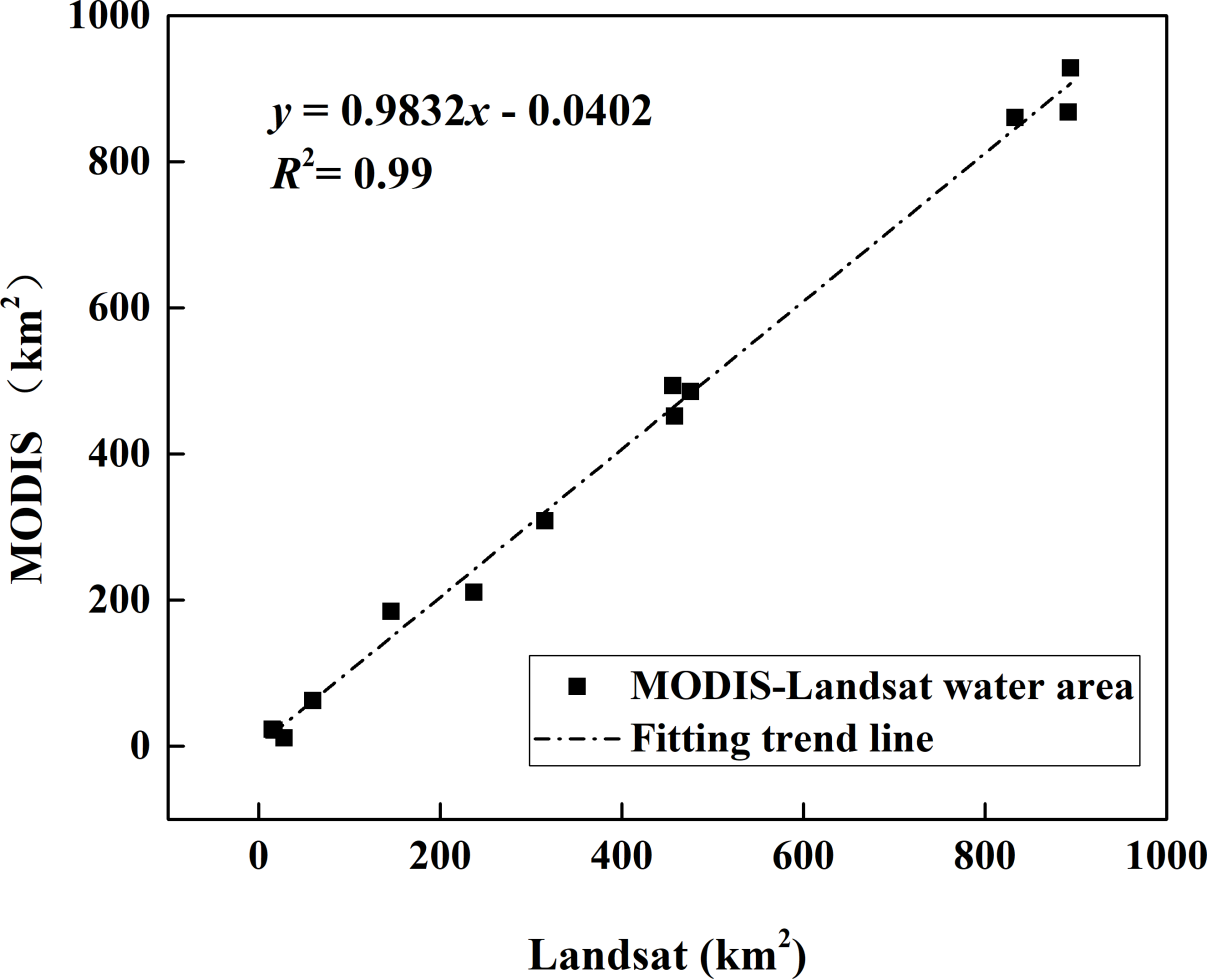
The linear relationship between two classification results.

The lake surface area determined by MODIS and Landsat ETM+ is shown in Fig. 3 and can be used to compare the two classification results. The dark blue in the figure represents the surface area extracted by MODIS and the light blue represents the surface area extracted from Landsat images. There was a good agreement between the two classification results in spatial distribution. The linear relationship between MODIS and Landsat was established by least square method. There is a good correlation between the two kinds of surface area data (Fig. 4) (*R*^2^ = 0.99, *P* < 0.01).

#### Water level

**Figure 5:**
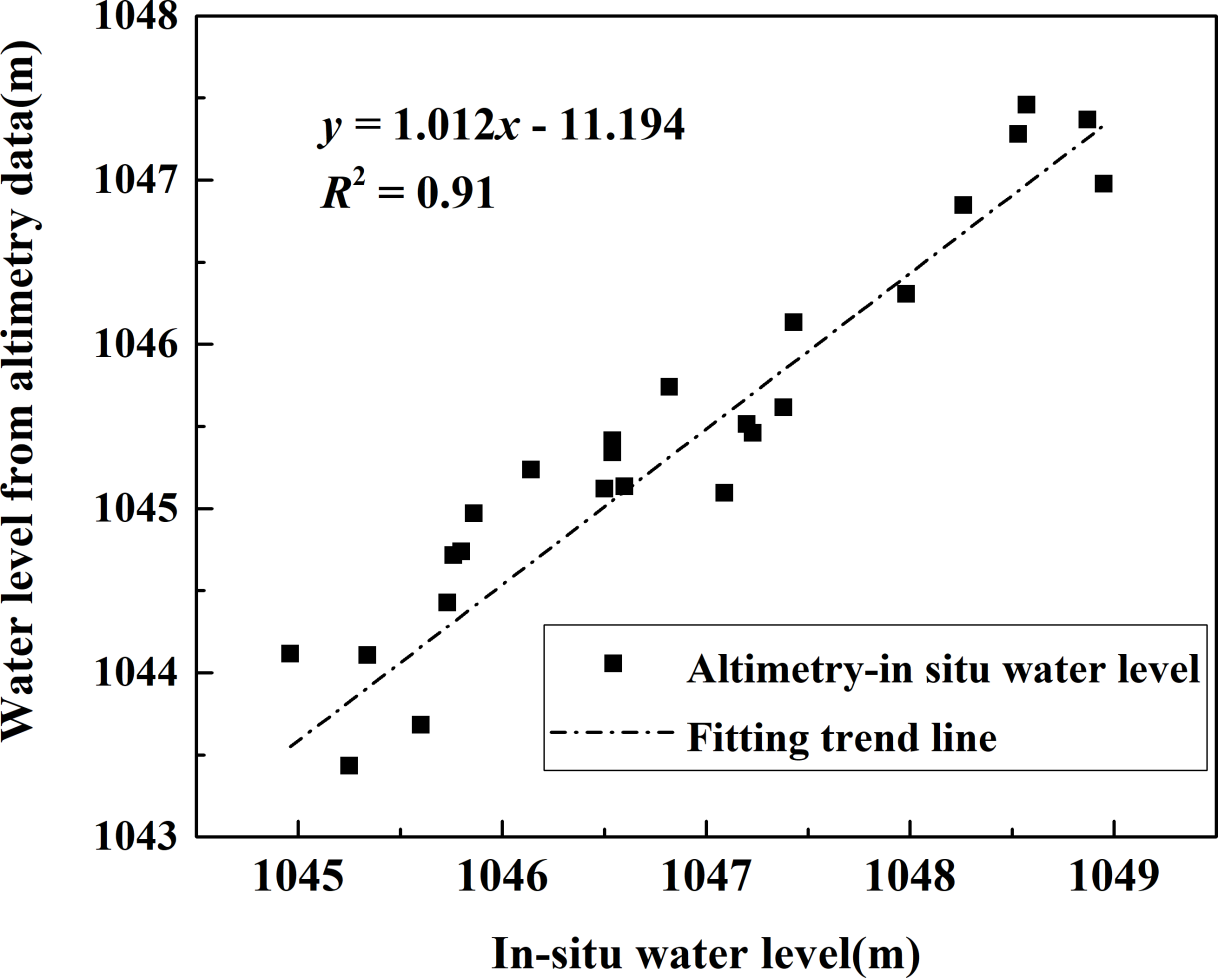
Comparison of water levels from satellite altimetery and in situ measurement.

In order to validate the water level, we obtained monthly in situ measurements of the water level of Bosten Lake from 2002 to 2010 and compared it with the satellite altimeter results. The results show that the time-series water level from the altimetry data was in good agreement with in situ measurements (Fig. 5). There was a high correlation between the measured data with our altimeter results and the correlation coefficient was 0.91 (*P* < 0.01). Envisat/RA-2 and ICESat/GLAS altimetry data can be feasibly and reliably combined to monitor the changes of lake water levels in Xinjiang.

#### Changes of surface area

**Figure 6:**
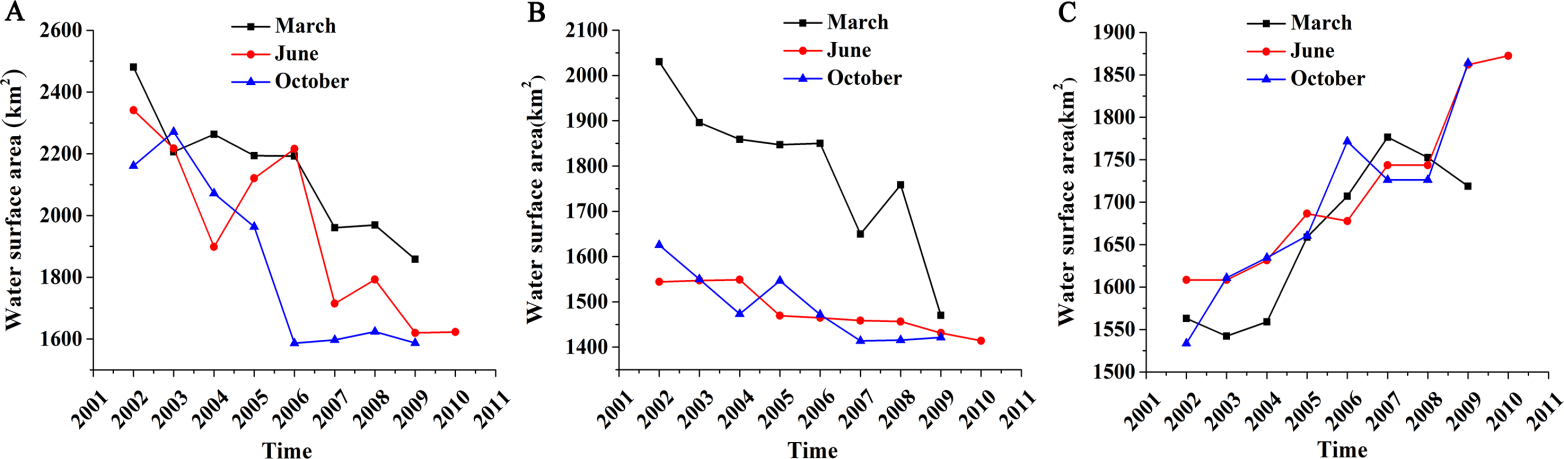
Total water surface area changes of different altitude lakes in different months. (A) Low-altitude lakes; (B) medium-altitude lakes; (C) high-altitude lakes.

We calculated the total area of 13 lakes and analyzed their annual and inter-annual changes (Fig. 6). The changes in the area of the lake surfaces was quite large. The overall trend showed that the lakes in low- and medium-altitude areas were shrinking but lakes in high-altitude areas were expanding. The largest surface area of lakes in low- and medium-altitude areas was typically seen in March and the smallest surface area was seen in October. Lakes were at their lowest levels in 2007. However, there was no significant monthly difference in the surface area of the lakes in the high-altitude areas.

There were five lakes at low-altitude (Ebinur, Ailike, Jili, Ulungur, and Manas) and each showed a significantly decreasing trend for water area in March, June, and October from 2002 to 2010. The change in rate for June (30.69%) was the largest relative to other months. The total water area in June 2002 was approximately 2,341.39 km^2^ but shrank to 1,622.73 km^2^ in June 2010. The rates of change in March and October were 25.08% and 26.57%, respectively.

Four lakes were studied in the medium-altitude area (Bosten, Sayram, Salt, and Chaiwopu). The total water area showed a decreasing trend but it was not as drastic as in the low-altitude area. The total water area was approximately 1,544.25 km^2^ in June 2002, but decreased to 1,414.17 km^2^ in June 2010. There was a drastic change in March, with a rate of change of 28.64%. The rates of change in June and October were relatively small (8.42% and 12.57%, respectively).

Four large lakes were studied in the high-altitude area (Aksaichin, Aqqikkol, Ayakkum, and Jingyu) and all of them showed a significantly increasing trend. The total water area was approximately 1,608.44 km^2^ in October 2002 and expanded to 1,872.47 km^2^ in June 2010. The rates of change were 9.96%, 16.42% and 21.53% in March, June, and October, respectively. No lakes were identified in extremely high-altitude areas (above 5,000 m) and were not included in this study.

#### Changes of water level

**Figure 7:**
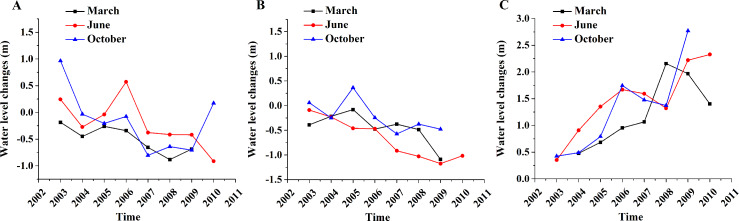
Mean water level changes in different altitudes and months. (A) Low-altitude lakes; (B) medium-altitude lakes; (C) high-altitude lakes.

The changes of water level at different altitudes were very similar to the changes in water surface area. However, there were some distinctions (Fig. 7): First, the monthly distinction of water level in low- and medium-altitudes was much smaller than that of the surface area. Secondly, the change in water levels reached a relatively high point in June and October of 2006 in low- and high-altitude areas, but were found in March and October of 2005 in medium-altitude areas. The water level tended to decrease in low- and medium-altitude areas and increase in high-altitude areas. The water level tended to decrease in March (−0.10 m/year) and increase in June (0.03 m/year) and October (0.04 m/year).

### Relationship of lake surface area and lake water level

**Figure 8:**
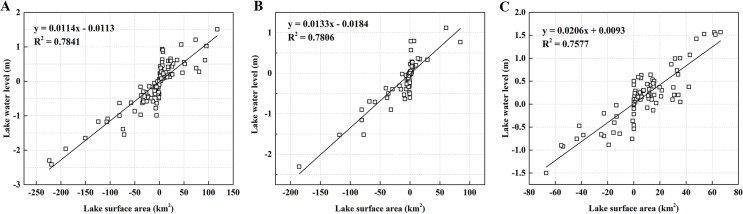
The correlation between lake surface area and water level in different altitude. (A) Low-altitude lakes; (B) medium-altitude lakes; (C) high-altitude lakes.

Lake water level is one of the important measurements linking lake area and water volume. We conducted correlation analysis to observe the relationship between lake surface area and water level changes in Xinjiang. The correlation of water surface area and water level of lakes at different altitudes from 2002 to 2010 is shown in Fig. 8. The correlation coefficients reveal a close relationship with *R*^2^ = 0.76 (*P* < 0.01), indicating that the lake level changes were closely correlated with the changes of lake surface area.

The relationship between the lake water levels and surface areas suggests the high reliability of the extrapolated lake water levels based on lake surface area and empirical models. We estimated the change of lake water volume from 2011 to 2018 using the water level data from the empirical model.

### Water volume changes

**Figure 9:**
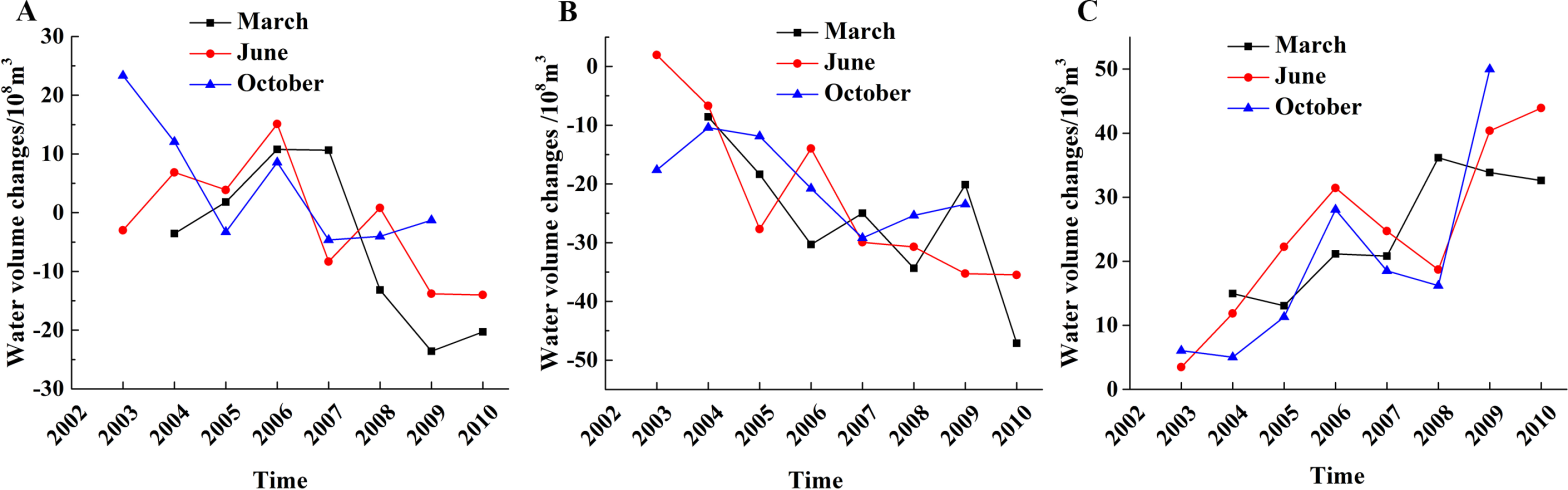
Water volume changes of different altitude lakes in different months. (A) Low-altitude lakes; (B) medium-altitude lakes; (C) high-altitude lakes.

We estimated the relative water volume changes of two adjacent periods from 2002 to 2010 using the water volume estimation model. The cumulative changes of lake water volume were consistent with the changes of lake surface area and water level. There was a decreasing trend in low- and medium-altitude areas, but an increasing trend in the high-altitude area (Fig. 9). Lake volumes decreased in March (37.55×10^8^ m^3^) but increased in June (3.48×10^8^ m^3^) and October (26.90×10^8^ m^3^).

The water volume decreased by −24.16 ×10^8^ m^3^, −15.05 ×10^8^ m^3^ and 3.12 ×10^8^ m^3^ in March, June, and October, respectively, in the low-altitude area. Water volume generally increased before 2006 and began a rapid decline after 2006. The most pronounced declines occurred in March and June.

The change in water volumes in the medium-altitude areas showed an obvious decreasing trend of −46 ×10^8^ m^3^, −35.51 ×10^8^ m^3^ and −26.94 ×10^8^ m^3^ in March, June, and October, respectively.

The water volume in the high-altitude area showed the largest increase. However, the changes between 2006 and 2008 fluctuated with increases before and after 2006. The first decrease took place after 2006 and increased again after 2008.The specific increasing amount was 32.61×10^8^ m^3^, 43.92×10^8^ m^3^ and 50.71×10^8^ m^3^ in March, June and October during 2002 to 2010.

### Spatial changes of water volume and level

**Figure 10:**
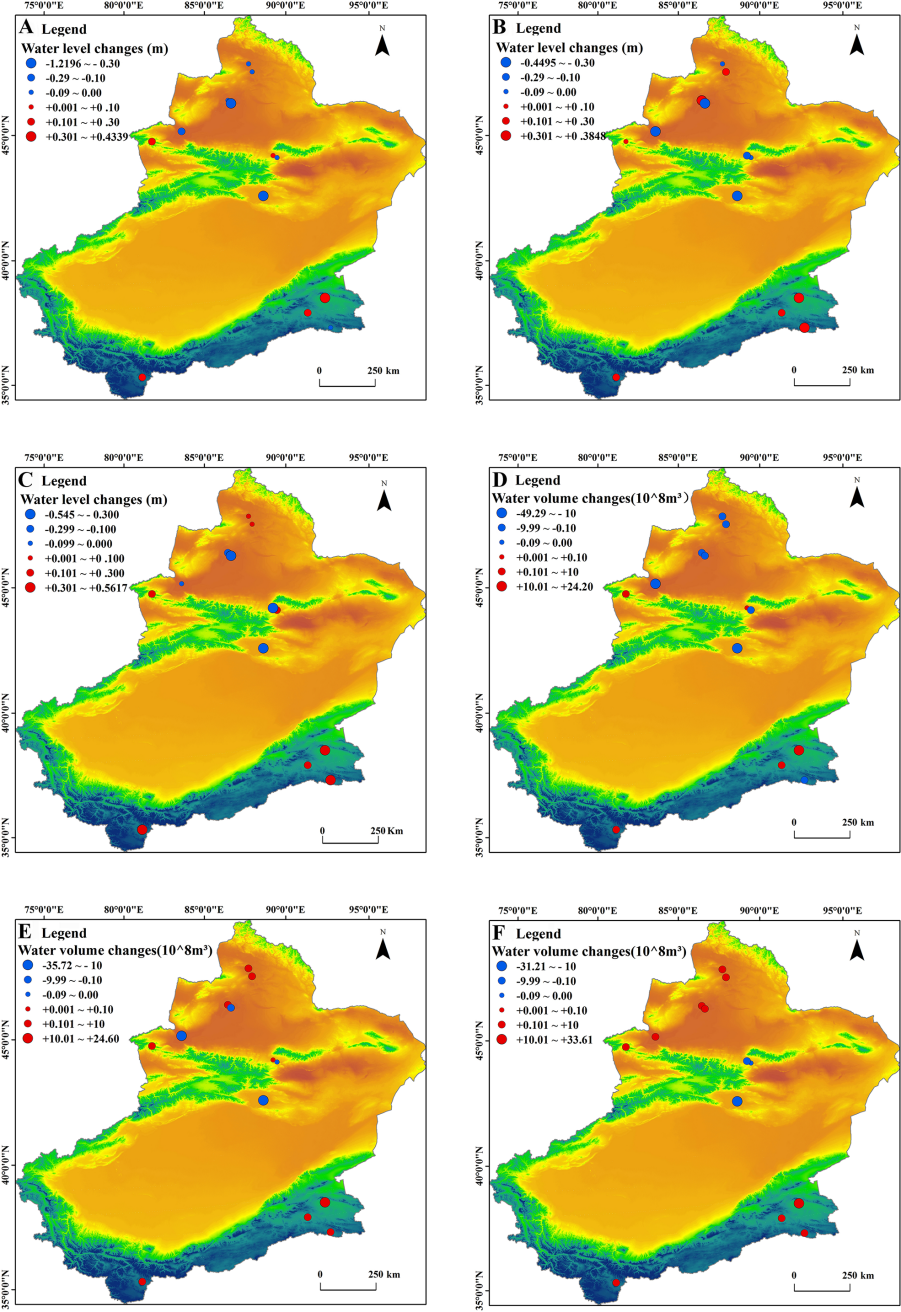
Changing trends of lake water level and volume in Xinjiang. (A-C) Changes of water level in March, June and October; (D-F) was the changes of water volume in March, June and October.

Ebinur Lake has the largest change of the low-altitude lakes with water volume changes in March, June, and October of −18.29 ×10^8^ m^3^, −18.06×10^8^ m^3^, and 0.62×10^8^ m^3^. The water level changes were −0.27, −0.30, and −0.004 m/year, respectively. The water level changes of Ulungur Lake were extremely small and were generally stable in different months (−0.02, 0.06, 0.01 m/year) (Fig. 10).

The dramatic decrease of Bosten Lake’s water volume marked the greatest decline in the medium-altitude area. The water volume changes of Bosten Lake were −49.29 ×10^8^ m^3^, −35.72 ×10^8^ m^3^, and −31.21 ×10^8^ m^3^ and the water level changes were −0.56, −0.45, and −0.41 m/year in March, June, and October, respectively. Chaiwopu Lake had the smallest changes in water volume and levels compared to other lakes. The changes in water volume were 0.07 ×10^8^ m^3^, 0.02 ×10^8^ m^3^ and −0.11 ×10^8^ m^3^ and the changes in water level were 0.03, −0.25, −0.54 m/year.

The water volume and levels of the four lakes in the high-altitude area increased significantly in all months, with the exception of Jingyu Lake in March (−0.2 ×10^8^ m^3^, −0.01 m/year). The changes of Ayakkum Lake were more pronounced than other lakes and the water volume changes were 24.20 ×10^8^ m^3^, 24.63 ×10^8^ m^3^, and 33.61 ×10^8^ m^3^ in March, June, and October, respectively.

### Cumulative changes of lake volume in 16 years

**Figure 11:**
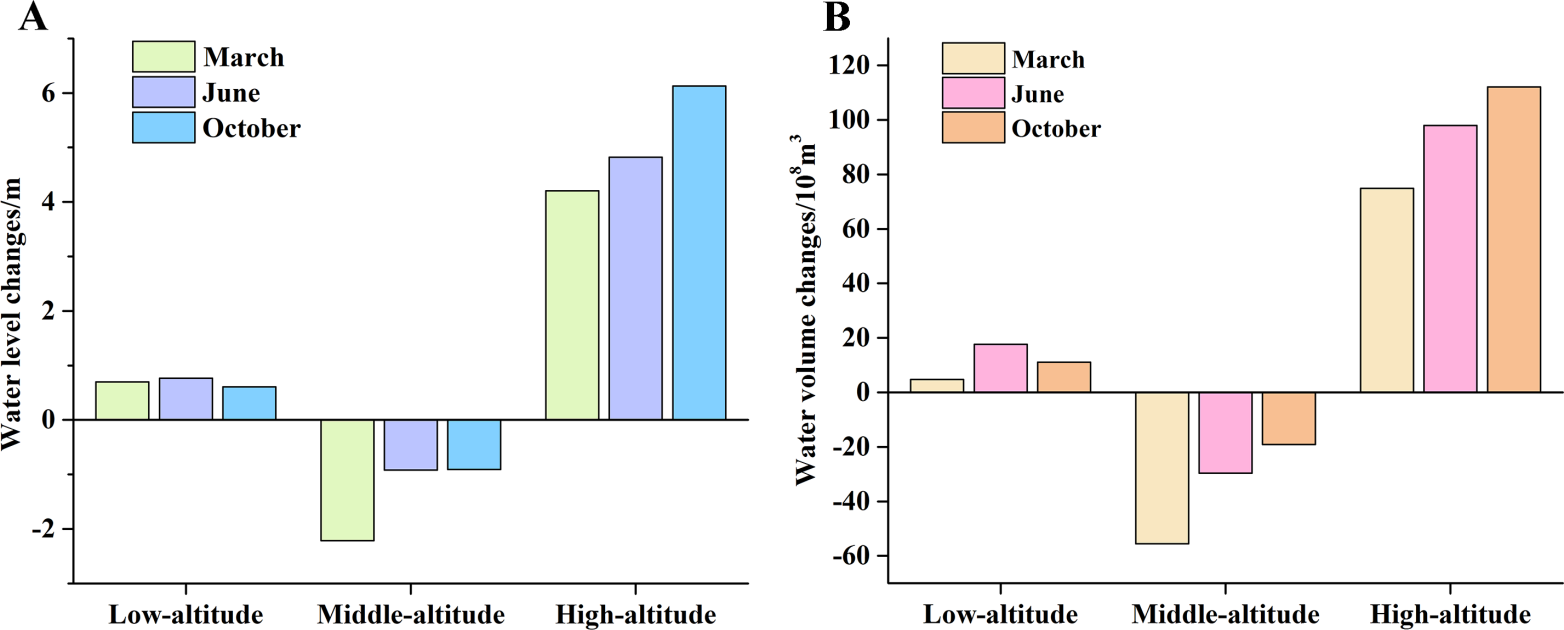
The changes of water level (A) and water volume (B) in different months from 2002 to 2018.

The cumulative changes of lake water level and water volume during 2002–2018 (Fig. 11) indicated a similar trend in water levels and volume. The water level and volume tended to increase in areas of low-altitude but decreased at a medium-altitude. However, water level and water volume significantly increased at high-altitude.

There was no significant difference in water level changes in the lakes at low-altitudes in March, June, or October. The total increase of the water level in all lakes was less than 1 m (Fig. 11A). Water volume increased significantly in June (17.62 ×10^8^ m^3^) but very little in March (4.69 ×10^8^ m^3^), indicating a significant seasonal difference (Fig. 11B).

Most of the lakes in the low-altitude area were relatively flat so the changes of water surface area were more dramatically visualized than the changes in water level. As a result, there was a small change in water level but a large change in water volume.

The changes of water level and water volume were similar in the medium-altitude area, with a significant decline in both. The decline was most obvious in March (−2.2 m, −55.57 ×10^8^ m^3^), followed by June (0.92 m, −29.67 ×10^8^ m^3^) and October (0.91 m, −19.08 ×10^8^ m^3^). The total water level in the medium-altitude area decreased by more than 2 m and the total water volume fell by more than 55 ×10^8^ m^3^.

The water level and water volume increased dramatically in the high-altitude area. The total water level of all lakes increased by more than 6 m from 2002 to 2018 and the total water volume exceeded 110 ×10^8^ m^3^; the largest increase was in October (6.13 m, 112.14 ×10^8^ m^3^), followed by June (4.28 m, 97.95 ×10^8^ m^3^), and March (4.21 m, 74.86 ×10^8^ m^3^), with significant seasonal differences.

## Discussion

### The increase of water volume in high-altitude areas

**Figure 12:**
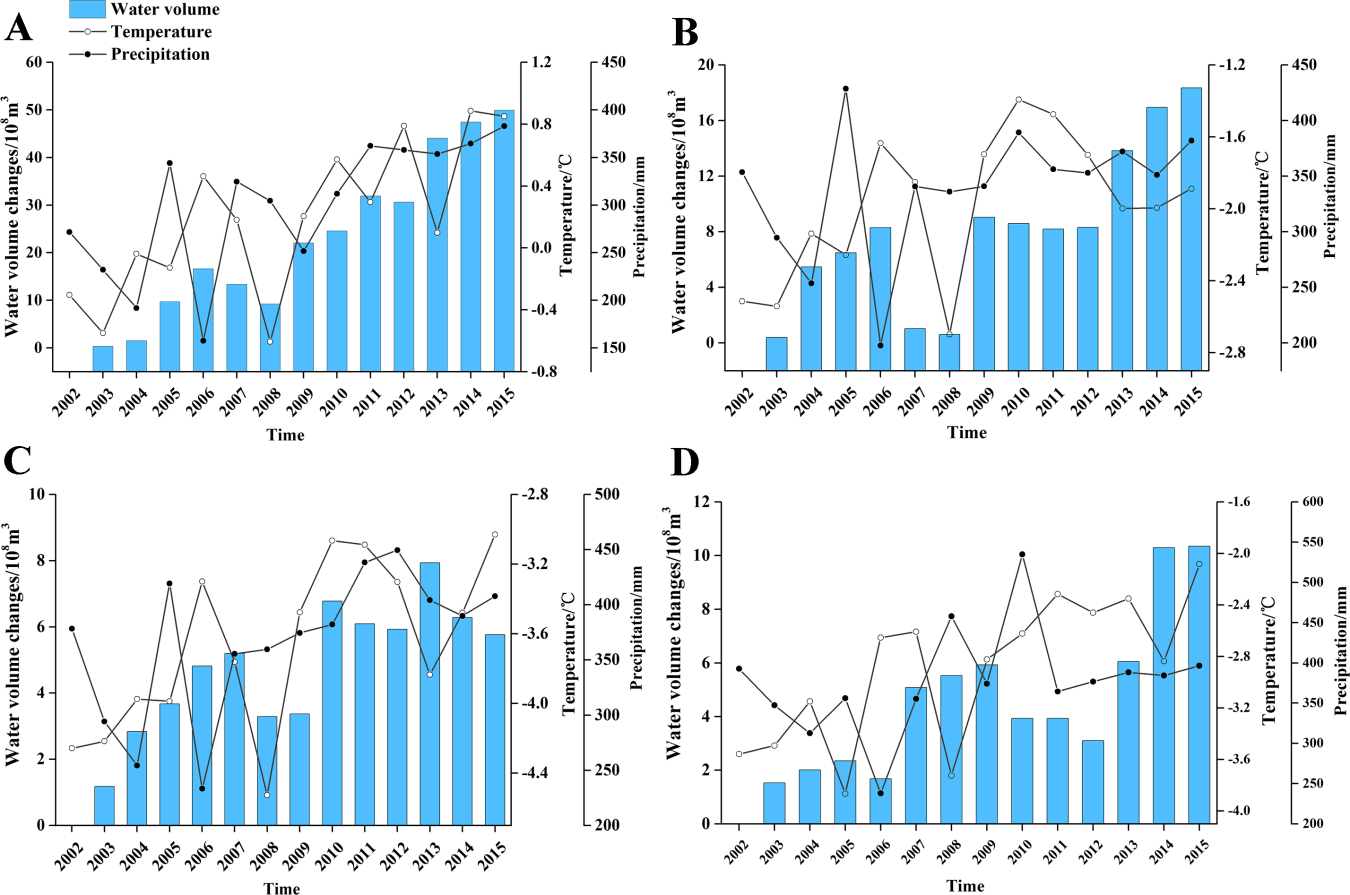
The changes of water volume and climate factors in high-altitude lakes. (A) Ayakkum Lake, (B) Aqqikkol Lake, (C) Jingyu Lake, (D) Aksaichin Lake.

The changes noted in lakes at different altitudes were due to the different climatic characteristics and intensities and patterns of human activities. The temperature and precipitation of four lakes showed a slight increasing trend from 2002 to 2015 (Fig. 12), consistent with the increasing trend of water volume. The climate of the arid region of northwest China increased consistently with climate changes. The rate of temperature change in the northwest region over the last 50 years was as high as 0.368 °C. This was 10a^−1^ higher than the Chinese average and the global warming trend (IPCC, 2007), which was 1.31 times China’s average rate in rising temperature and 2.62 times the global average rate of temperature rise. The temperature increase was even greater after 2000, with an average annual temperature increase of 1.0 °C. The lakes absorbed an abundance of precipitation and contributed to the wet island effect in the arid northwest region because of the high-altitude and topographic uplift. The increase in temperature and precipitation may have contributed to the increase of water volume of these high-altitude lakes.

Glacial meltwater may also contribute to the increase in water volume. The volumes of the Ayakkum, Aqqikkol, Jingyu, and Aksaichin lakes were controlled by the continental desert climate of the plateau, were less affected by human activities (Bian et al., 2006), and were mainly dependent on the runoff from snowmelt water. A global temperature rise may have contributed to an increase in snow and ice melt replenishing those lakes, leading to increase of water volume.

**Figure 13:**
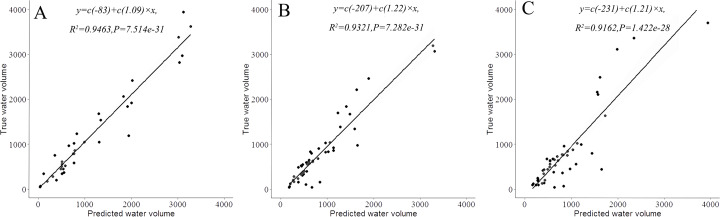
Random forest regression model for water volume and climate factors in high altitude lakes. (A) March, (B) June, (C) October.

We established a random forest regression model for the lakes in the high-altitude area to determine the contributions of climate (temperature and precipitation) to the changes of lake water volumes in different months. The results showed that the R^2^ were 0.94 in March, 0.93 in June, and 0.92 in October. All models met the condition of *P* < 0.001 (Fig. 13). The variable importance of temperature and precipitation were 31.84% and 15.97% in March 33.97% and 18.23% in June, and 30.05% and 16.75% in October. These results revealed that temperature and precipitation can influence lake water volume, but that their contributions were not high, with temperature contributing about 30% and precipitation about 15%. The dynamic changes of the lake are closely related to water surface evaporation, surface runoff, melting water, groundwater recharge and other factors according to the water balance principle. We did not consider other factors because there was no previously measured data for high-altitude lakes.

### Possible future of lakes in high-altitude area of arid region

Rising temperatures have increased the water volume of high-altitude lakes since 2002 and the continued warming of the climate could cause the total melt of mountain glaciers. Therefore, the changes of high-altitude lake volumes may be similar to those seen in low-altitude lakes. Water from glacier and snow melt may cause changes sequentially seen as: (1) large glaciers become thinner, (2) small and mid-sized glaciers disappear, and (3) snowlines recede. According to previous research, glaciers in high mountains in Xinjiang were already retreating with warmer temperatures (He et al., 2019; Li et al., 2019). 2 of 8 typical glaciers in the middle Tianshan mountains retreated at a mean rate of < 5 m/year between 1963 and 1986, and between 1986 and 2000, seven of those glaciers retreated at a mean rate of 10–15 m/year (Sun & W, 2006). The snow cover area in Xinjiang has declined significantly in recent years (Chen, Li & Hui, 2017; Haidi, Zhongqin & Mingjun, 2018). The water surface area, water depth, and water volume of lakes at high-altitudes may see a decline in the future as mountain glaciers and snow continue to melt.

### Satellite altimetry limitations

Larger lakes were more frequently captured by satellites due to the low coverage limitations of altimetry data. The integration of two kinds of satellite altimetry data were necessary to obtain an accurate water level of all target lakes and to better analyze medium and small lakes.

Altimetry measurements can range in accuracy from a few centimeters to tens of centimeters. The accuracy of the measurements is determined by several factors including the size and shape of target, the complexity of the surrounding topography, winter ice cover, and surface waves (Crétaux et al., 2017). The factors limit the use of satellite altimetry data in small-to-medium-scale bodies of water and may have affected the accuracy of the measurement of small lakes like Chaiwopu Lake and Salt Lake. The temporal resolution of satellite altimeter data can be greatly limited and its short time span cannot satisfy the needs to measure long periods of variation.

## Conclusions

Xinjiang has a complex terrain and distinct climate at different altitudes. Determining water volume changes at different altitude area is essential for assessing the effects of climate change and human activities on lakes. We studied the water volume of 13 lakes at different altitudes in Xinjiang and estimated their water content based on MODIS and altimetry data. The relationship of water volume changes with climate change and was explored. We determined that:

(1) The changes in lake surface areas, levels, and volumes between different altitudes were quite large. The lakes in low- and medium-altitude areas shrank between 2002–2010 but lakes at high-altitudes expanded. The lake water levels tended to decrease in March (−0.10 m/year) but increase in June (0.03 m/year) and October (0.04 m/year). The lake level changes were closely correlated with lake surface area changes (*R*^2^ = 0.74, *P* < 0.01).

(2) The changes of water volume were consistent with the changes in water level, decreasing in March (37.55 × 10^8^ m^3^), but increasing in June (3.48 ×10^8^ m^3^) and October (26.90 × 10^8^ m^3^). A time series of lake water volume was reconstructed from 2011 to 2018 based on the empirical model, and the total lake water volume exhibited a slightly increasing trend during 2011–2018 (71.35 ×10^8^ m^3^). Changes in lake water in high-altitude areas are thought to be dominated by temperature-induced glacial snow melt. Lakes in low- and medium-altitude areas are more influenced by human activities like cultivated land expansion-induced agricultural irrigation. These results are based on the analysis of regional precipitation, and temperature and lake water change in specific months.

##  Supplemental Information

10.7717/peerj.9683/supp-1 Supplemental Information 1 Hydroweb data The raw data was used to further verify the accuracy of SVM classification results.

## Additional Information and Declarations

### Data Availability

The following information was supplied regarding data availability: The raw data are available in the Supplemental File.

## References

[ref-1] Baup F, Frappart F, Maubant J (2014). Combining high-resolution satellite images and altimetry to estimate the volume of small lakes. Hydrology and Earth System Sciences.

[ref-2] Bhang KJ, Schwartz FW, Braun A (2007). Verification of the vertical error in C-band SRTM DEM using ICESat and Landsat-7, Otter Tail County, MN. Ieee Transactions on Geoscience and Remote Sensing.

[ref-3] Bian D, Yang Z, Li L, Chu D, Zhuo G (2006). The response of lake area change to climate variations in North Tibetan Plateau during last 30 years. Acta Geographica Sinica.

[ref-4] Busker T, de Roo A, Gelati E, Schwatke C, Adamovic M, Bisselink B, Pekel J-F, Cottam A (2019). A global lake and reservoir volume analysis using a surface water dataset and satellite altimetry. Hydrology and Earth System Sciences.

[ref-5] Chen L, Li Z, Hui Z (2017). Temporal and spatial distribution of snow cover in Altai region, Xinjiang from 2001 to 2014. Journal of Arid Land Resources and Environment.

[ref-6] Chipman J (2019). A multisensor approach to satellite monitoring of trends in lake area, water level, and volume. Remote Sensing.

[ref-7] Crétaux JF, Nielsen K, Frappart F, Papa F, Calmant S, Benveniste J (2017). Hydrological applications of satellite altimetryrivers, lakes, man-made reservoirs, inundated areas. satellite altimetry over oceans and land surfaces.

[ref-8] Dai X, Yang X, Wang M, Gao Y, Liu S, Zhang J (2020). The dynamic change of bosten lake area in response to climate in the past 30 years. Water.

[ref-9] Davranche A, Lefebvre G, Poulin B (2010). Wetland monitoring using classification trees and SPOT-5 seasonal time series. Remote Sensing of Environment.

[ref-10] Dilinuer A, Aikebaer (2010). Study on dynamic change of main lakes water area in Xinjiang. Journal of China Hydrology.

[ref-11] Frappart F, Biancamaria S, Normandin C, Blarel F, Darrozes J (2018). Influence of recent climatic events on the surface water storage of the Tonle Sap Lake. Science of the Total Environment.

[ref-12] Frappart F, Calmant S, Cauhope M, Seyler F, Cazenave A (2006). Preliminary results of ENVISAT RA-2-derived water levels validation over the Amazon basin. Remote Sensing of Environment.

[ref-13] Gardini B, Graf G, Astronautica GRJA (1995). The instruments on envisat. Acta Astronautica.

[ref-14] Hanqiu X (2007). Modification of normalised difference water index (NDWI) to enhance open water features in remotely sensed imagery. International Journal of Remote Sensing.

[ref-15] Haidi H, Zhongqin L, Mingjun Z (2018). Spatio-temporal variation analysis of snow cover area of Tianshan Mountains in China using MODIS data. Arid Land Geography.

[ref-16] He J, Wang N, Chen Aa, Yang X, Hua T (2019). Glacier changes in the qilian mountains, northwest China, between the 1960s and 2015. Water.

[ref-17] IPCC (2007). climate change the physical science basis.

[ref-18] Ji L, Geng X, Sun K, Zhao Y, Gong P (2015). Target detection method for water mapping using landsat 8 OLI/TIRS imagery. Water.

[ref-19] Khaki M, Awange J, Forootan E, Kuhn M (2018). Understanding the association between climate variability and the Nile’s water level fluctuations and water storage changes during 1992-2016. Science of the Total Environment.

[ref-20] Khandelwal A, Karpatne A, Marlier ME, Kim J, Lettenmaier DP, Kumar V (2017). An approach for global monitoring of surface water extent variations in reservoirs using MODIS data. Remote Sensing of Environment.

[ref-21] Laxon S (1994). Sea ice altimeter processing scheme at the EODC. International Journal of Remote Sensing.

[ref-22] Legrésy B, Remy F (1997). Altimetric observations of surface characteristics of the Antarctic ice sheet. Journal of Glaciology.

[ref-23] Lei J, Li Z, Bruce W (2009). Analysis of dynamic thresholds for the normalized difference water index. Photogrammetric Engineering & Remote Sensing.

[ref-24] Li L, Yao X, Liu S, Bu Y, Gong P, Li X (2019). Glacier changes along the Silk Road Economic Belt in China in the past 50 years. Journal of Natural Resources.

[ref-25] Li X, Jiang F, Li L, Wang G (2011). Spatial and temporal variability of precipitation concentration index, concentration degree and concentration period in Xinjiang, China. International Journal of Climatology.

[ref-26] Medina C, Gomez-Enri J, Alonso JJ, Villares P (2010). Water volume variations in Lake Izabal (Guatemala) from in situ measurements and ENVISAT Radar Altimeter (RA-2) and Advanced Synthetic Aperture Radar (ASAR) data products. Journal of Hydrology.

[ref-27] Mountrakis G, Im J, O C (2011). Support vector machines in remote sensing: a review. Isprs Journal of Photogrammetry & Remote Sensing.

[ref-28] Ouma OY, Tateishi R (2007). A water index for rapid mapping of shoreline changes of five East African Rift Valley lakes: an empirical analysis using Landsat TM and ETM+ data. International Journal of Remote Sensing.

[ref-29] Pal M, Foody GM (2010). Feature selection for classification of hyperspectral data by SVM. IEEE Transactions on Geoscience & Remote Sensing.

[ref-30] Peng D, Guo S, Liu P (2006). Reservoir storage curve estimation based on remote sensing data. Journal of Hydrologic Engineering.

[ref-31] Qiao B, Zhu L, Yang R (2019). Temporal-spatial differences in lake water storage changes and their links to climate change throughout the Tibetan Plateau. Remote Sensing of Environment.

[ref-32] Ricko M, Carton JA, Birkett CM, Crétaux JF (2012). Intercomparison and validation of continental water level products derived from satellite radar altimetry. Journal of Applied Remote Sensing.

[ref-33] Sarp G, Ozcelik M (2016). Water body extraction and change detection using time series: a case study of Lake Burdur, Turkey. Journal of Taibah University for Science.

[ref-34] Schindler D (2009). Lakes as sentinels and integrators for the effects of climate change on watersheds, airsheds, and landscapes. Limnology and Oceanography.

[ref-35] Schutz BE, Zwally HJ, Shuman CA, Hancock D (2005). Overview of the icesat mission. Geophysical Research Letters.

[ref-36] Song C, Ye Q, Sheng Y, Gong T (2015). Combined ICESat and CryoSat-2 altimetry for accessing water level dynamics of Tibetan lakes over 2003–2014. Water.

[ref-37] Sun ZD, W R (2006). Effect of glaciers change to water balance of Lake Bosten under climatic backgrounds.

[ref-38] Taube CM (2000). Instructions for winter lake mapping. Chapter 12. Manual of fisheries survey methods ii: with periodic updates.

[ref-39] Tong X, Pan H, Xie H, Xu X, Li F, Chen L, Luo X, Liu S, Chen P, Jin Y (2016). Estimating water volume variations in Lake Victoria over the past 22 years using multi-mission altimetry and remotely sensed images. Remote Sensing of Environment.

[ref-40] Vermote E (2015). MOD09A1 MODIS surface reflectance 8-Day L3 Global 500m SIN Grid V006.

[ref-41] Wang J, Ding J, Li G, Liang J, Yu D, Aishan T, Zhang F, Yang J, Abulimiti A, Liu J (2019). Dynamic detection of water surface area of Ebinur Lake using multi-source satellite data (Landsat and Sentinel-1A) and its responses to changing environment. Catena.

[ref-42] Wang L, Gong H, Shao Y (2014). Precise topography assessment of Lop Nur Lake Basin using GLAS altimeter. IOP Conference Series: Earth and Environmental Science.

[ref-43] Wang T, Yan CZ, Song X, Xie JL (2012). Monitoring recent trends in the area of aeolian desertified land using Landsat images in China’s Xinjiang region. ISPRS Journal of Photogrammetry and Remote Sensing.

[ref-44] Wang X, Cheng X, Gong P, Huang H, Li Z, Li X (2011). Earth science applications of ICESat/GLAS: a review. International Journal of Remote Sensing.

[ref-45] Williamson CE, Saros JE, Schindler DW (2009). CLIMATE CHANGE: sentinels of change. Science.

[ref-46] Wingham DJ, Rapley CG, Griffiths HD (1986). New techniques in satellite altimeter tracking systems. IGARSS 86 Symposium ESA SP-.

[ref-47] Wu S, Zhang Q (2005). Method and model of water body extraction based on remote sensing data of MODIS. Computer & Digital Engineering.

[ref-48] Yang J, Ma L, Li C, Liu Y, Ding J, Yang S (2019). Temporal-spatial variations and influencing factors of Lakes in inland arid areas from 2000 to 2017: a case study in Xinjiang. Geomatics Natural Hazards and Risk.

[ref-49] Zhang F, Johnson VC, Kung HT, Ding JL, Sun Q, Zhou M, Kelimu A, Nurmuhammat I, Chan NW (2015a). The influence of natural and human factors in the shrinking of the Ebinur Lake, Xinjiang, China, during the 1972–2013 period. Environ Monit Assess.

[ref-50] Zhang G, Chen W, Xie H (2019a). Tibetan plateau’s lake level and volume changes from NASA’s ICESat/ICESat-2 and landsat missions. Geophysical Research Letters.

[ref-51] Zhang G, Xie H, Yao T, Kang S (2013). Water balance estimates of ten greatest lakes in China using ICESat and Landsat data. Chinese Science Bulletin.

[ref-52] Zhang G, Yao T, Chen W, Zheng G, Shum CK, Yang K, Piao S, Sheng Y, Yi S (2019b). Regional differences of lake evolution across China during 1960s–2015 and its natural and anthropogenic causes. Remote Sensing of Environment.

[ref-53] Zhang Q, Sun P, Li J, Singh VP, Liu J (2015b). Spatiotemporal properties of droughts and related impacts on agriculture in Xinjiang, China. International Journal of Climatology.

[ref-54] Zhao Y, Liao JJ, Shen GZ, Zhang XL (2017). Monitoring lake level changes by altimetry in the arid region of Central Asia. IOP Conference Series: Earth and Environmental Science.

[ref-55] Zhou C, Cheng W, Qian J, Li B, Zhang B (2010). Research on the Classification System of Digital Land Geomorphology of 1:1 000 000 in China. Geo-information Science.

[ref-56] Ziyinali H (2018). Lake changes in spatial evolution and driving force for the water area change of the Manas Lake in Xinjiang in the past forty years. Remote Sensing for Land and Resources.

[ref-57] Zuo Q, Dou M, Chen XI, Zhou K (2006). Physically-based model for studying the salinization of Bosten Lake in China. Hydrological Sciences Journal.

[ref-58] Zwally HJ, Schutz B, Abdalati W, Abshire J, Bentley C, Brenner A, Bufton J, Dezio J, Hancock D, Harding D, Herring T, Minster B, Quinn K, Palm S, Spinhirne J, Thomas R (2002). ICESat’s laser measurements of polar ice, atmosphere, ocean, and land. Journal of Geodynamics.

